# Announcement

**DOI:** 10.1155/S1463924698000194

**Published:** 1998


Journal of Automatic Chemistry, Vol. 20, No. 4 (July–August 1998) p. 129

### Announcement

### ISLAR'98—International Symposium on Laboratory Automation and Robotics

*Preliminary programme*

ISLAR'98, the sixteenth in a continuing series of Symposia dedicated to laboratory automation and robotics, will be held at the Boston Park Plaza Hotel from 18 to 21 October 1998. A strong emphasis will be placed on presentations by senior laboratory managers on applications in drug discovery research, pharmaceutical and QC analysis, bioanalytical techniques, and chemical analysis.

The three-day programme consists of over 100 podium and poster presentations describing laboratory robotics and laboratory workstation procedures from pharmaceutical, biotechnology and chemical laboratories. The Symposium's technical presentations and informal discussions provide an excellent opportunity for laboratory managers, automation users and novices to exchange ideas and applications experience. In addition, there are six, free, laboratory-automation focused short courses this year. 1998 session topics include:

- combinatorial chemistry/process optimization
- automated structural characterization
- ultra-high throughput screening
- data management/data handling
- managing laboratory automation
- chemical analysis
- pioneer awardees: future trends in automation
- automated compound distribution drug discovery
- high throughput screening
- methods transfer and validation
- pharmaceutical analysis
- advanced topics
- bioanalytical assays

The opening session will feature three keynote speakers:

### Integrating drug discovery technologies

*J. A. Bristol, Parke-Davis Pharmaceutical Research, Ann Arbor, MI, USA*

The pharmaceutical industry is entering a new era wherein a number of new and enabling technologies such as combinatorial chemistry, ultra-high throughput screening, genomics, data management, etc. are maturing simultaneously. In addition, new and improved automation capabilities will have a dramatic impact on the optimization of these new technologies. This combination has the potential to transform pharmaceutical discovery into a highly efficient technologically advanced process which will demand appropriate resourcing and integration for optimal efficency.

As a Vice President at Parke-Davis, James A. Bristol is responsible for the Departments of Chemistry, Discovery Informatics and Compound Management.

### Coordinated development of multi-site/multi-functional analytical robotics applications: challenges and visions

*E. F. McNiff, Bristol-Myers Squibb, Princeton, NJ, USA*

The analytical development laboratory provides multiple opportunities for increasing productivity through automation and robotics. A challenge exists in maintaining the automation creativity of individual scientists while providing a mechanism for an integrated and coordinated approach within a multi-site, multi-functional organization. A wide range of automated systems have come from a diverse mix of scientists and engineers. An overview of these systems will be provided with an emphasis on the success and challenges of the various development approaches which were utilized.

Edward F. McNiff is Vice President of Analytical Research and Development where his primary responsibility is to provide direction and coordination of all aspects of analytical chemistry and analytical biochemistry.

### Quality control: organization, cycle times, compliance and efficiency

*J. M. Hawkins, Janssen Pharmaceutica, Titusville, NJ, USA*

Understanding the contributions that the laboratory can make in product/process development, process improvement, market surveillance, and general business is key to the pharmaceutical business today. Poor laboratory practice yields compliance issues, increased cost, increased cycle time and delayed product introductions. This talk will cover key areas of customer satisfaction, the role of the quality control and quality assurance laboratories, measures of accountability and progress, and an example of how laboratory robotics can help meet important goals.

Currently Vice President of QA at Janssen Pharmaceutica, Juanita M. Hawkins has 25 years experience in microbiology, sterilization, quality assurance and operations.

*For more information contact Christine O'Neil, ISLAR, 68 Elm Street, Hopkinton, MA 0178, USA. Tel.: 00 01 508 497 2224, fax: 00 01 508 435 3439, e-mail: islar@islar.com, website: www.islar.com*

0142-0453/98 $12·00 © 1998 Taylor & Francis Ltd

